# PPARβ/δ, a Novel Regulator for Vascular Smooth Muscle Cells Phenotypic Modulation and Vascular Remodeling after Subarachnoid Hemorrhage in Rats

**DOI:** 10.1038/srep45234

**Published:** 2017-03-22

**Authors:** Hongrong Zhang, Li Jiang, Zongduo Guo, Jianjun Zhong, Jingchuan Wu, Junchi He, Han Liu, Zhaohui He, Haitao Wu, Chongjie Cheng, Xiaochuan Sun

**Affiliations:** 1 Department of Neurosurgery, the First Affiliated Hospital of Chongqing Medical University, NO. 1 of Youyi Rd, Yuzhong District, Chongqing, China; 2 Department of Neurosurgery, the Affiliated Hospital of Zunyi Medical College, NO. 149 of Dalian Rd, Zunyi, Guizhou, China

## Abstract

Cerebral vascular smooth muscle cell (VSMC) phenotypic switch is involved in the pathophysiology of vascular injury after aneurysmal subarachnoid hemorrhage (aSAH), whereas the molecular mechanism underlying it remains largely speculative. Peroxisome proliferator-activated receptor β/δ (PPARβ/δ) has been implicated to modulate the vascular cells proliferation and vascular homeostasis. In the present study, we investigated the potential role of PPARβ/δ in VSMC phenotypic switch following SAH. Activation of PPARβ/δ by GW0742 and adenoviruses PPARβ/δ (Ad-PPARβ/δ) significantly inhibited hemoglobin-induced VSMC phenotypic switch. However, the effects of PPARβ/δ on VSMC phenotypic switch were partly obstacled in the presence of LY294002, a potent inhibitor of Phosphatidyl-Inositol-3 Kinase-AKT (PI3K/AKT). Furthermore, following study demonstrated that PPARβ/δ-induced PI3K/AKT activation can also contribute to Serum Response Factor (SRF) nucleus localization and Myocardin expression, which was highly associated with VSMC phenotypic switch. Finally, we found that Ad-PPARβ/δ positively modulated vascular remodeling in SAH rats, i.e. the diameter of basilar artery and the thickness of vessel wall. In addition, overexpression of PPARβ/δ by adenoviruses significantly improved neurological outcome. Taken together, this study identified PPARβ/δ as a useful regulator for VSMC phenotypic switch and vascular remodeling following SAH, providing novel insights into the therapeutic strategies of delayed cerebral ischemia.

Accumulated researches have explored the broad neuropathology following subarachnoid hemorrhage (SAH) though, it’s still poorly understood about the specific mechanism underlying delayed ischemic neurological deficit (DIND). Over the past decade, the dysfunction of cerebral vascular autoregulation was regarded as a critical contributor to the delayed cerebral ischemia, brain swelling and vasogenic edema^1^,^2^,^3^,^4^. As the basic structural and functional component of cerebral vascular neural network, vascular smooth muscle cells (VSMC) is essentially responsible for stabilization of vascular tone and regulation of blood flow^4^,^5^. Recent studies indicated a novel mechanism of VSMC phenotypic switch, which is believed to contribute to the vascular remodeling following SAH^6^,^7^.

As is known to all, the VSMC is highly specialized to express a unique repertoire of contractile proteins and maintain the physiologically perfusion homeostasis. However, in response to various cellular stimuli VSMC undergoes a profound transition from contractile to synthetic phenotype^8^,^9^, as indicated by increase of proliferation and synthesis of extracellular matrix components, leading to thickening of vessel wall or even stenosis of the lumen. Histologically, this kind of phenotypic switch was defined by the decreased expression of α-smooth muscle actin (α-SMA) and smooth muscle myosin heavy chain (SM-MHC), as well as the increase of osteopontin (OPN) and embryonic smooth muscle myosin heavy chain (Smemb) expression within VSMC^9^,^10^.

Peroxisome proliferator-activated receptor β/δ (PPARβ, referred to also as PPARδ) is a ligand activated receptor from the nuclear hormone receptor superfamily^11^, involved in diverse vascular disorders via the effects of anti-inflammatory, anti-apoptotic and vascular modulation^12^. For instance, activation of PPARβ/δ would attenuate the release of inflammatory factors in VSMC^13^. However, it’s still unclear about its molecular pathway induced by PPARβ/δ. Phosphatidyl-Inositol-3 Kinase-AKT (PI3K/AKT) signaling was well-documented in regulation of cellular proliferation, differentiation, migration and apoptosis. Recently, accumulative evidence showed that activation of PI3K/AKT pathway also participates in VSMC proliferation and migration^14^. In the current study, we proposed a role of PPARβ/δ in VSMC phenotypic switch and cerebral vascular autoregulation, potentially due to the involvement of PI3K/AKT signaling following SAH.

## Results

### Hemoglobin Induced Phenotypic Switch in Cultured Cerebral VSMC

**Figure 1:**
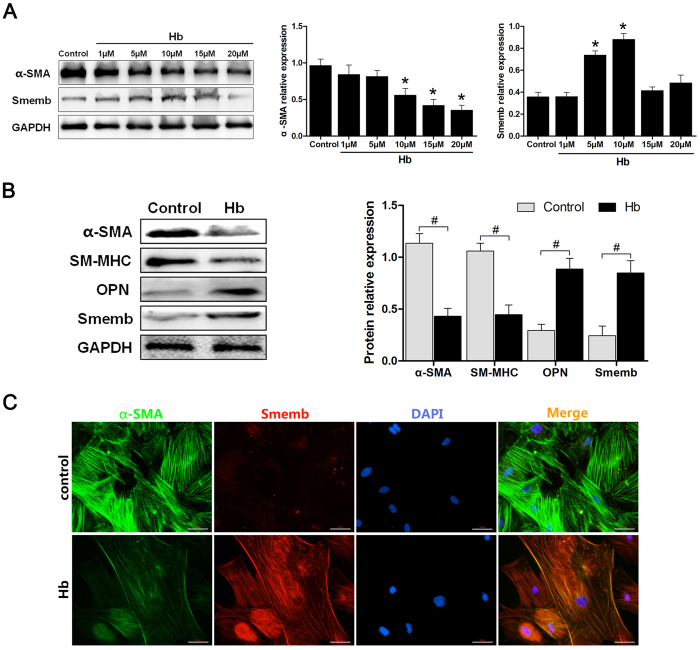
Expression profile of α-SMA, SM-MHC, Smemb and OPN in cerebral VSMC after treatment with Hb. (**A**) Representative image and quantitative analysis of α-SMA and Smemb expression in cerebral VSMC at 24 hours after incubation with different dosages of Hb respectively (n = 3 per group). (**B**) Representative image and quantitative analysis of α-SMA, SM-MHC, OPN and Smemb expression in cerebral VSMC at 24 hours after incubation with 10 μM Hb respectively (n = 3 per group). (**C**) Immunohistochemistry for α-SMA (green), Smemb (red), and DAPI (4′, 6-diamidino-2-phenylindole; blue) in cerebral VSMC at 24 hours after Hb incubation. All quantitative data were presented as mean ± standard deviation. Scale Bar = 50 μm. *P < 0.05 vs. Control; ^#^*P* < 0.05.

To validate the metabolic resource to initiate VSMC phenotypic switch following SAH, we treated the cells with different concentrations of hemoglobin, then the α-SMA and Smemb expression was examined by western blot after 24 hours. As shown in Fig. 1A, the α-SMA decrease and Smemb increase was detected within cultured VSMC after hemoglobin treatment. With the increased dose of hemoglobin concentration up to 10 μM, the α-SMA and Smemb expression exhibited maximal changes. However, excessive dose of hemoglobin resulted in the declined expression of both proteins probably due to cell collapse (Fig. 1A). According to the preliminary data, 10 μM (optimal stimulating concentration) was selected to perform subsequent experiments. After 24-hour incubation of hemoglobin, the expression of α-SMA and SM-MHC was significantly suppressed as compared to the control group. In contrast, it enhanced the expression of Smemb and OPN proteins (Fig. 1B). Likewise, after hemoglobin treatment decreased immunoreactivity of α-SMA and increased immunoreactivity of Smemb was observed within cultured VSMC (Fig. 1C).

### PPARβ/δ Agonist GW0742 Up-regulated PPARβ/δ Expression and Suppressed Cerebral VSMC Phenotypic Switch

**Figure 2:**
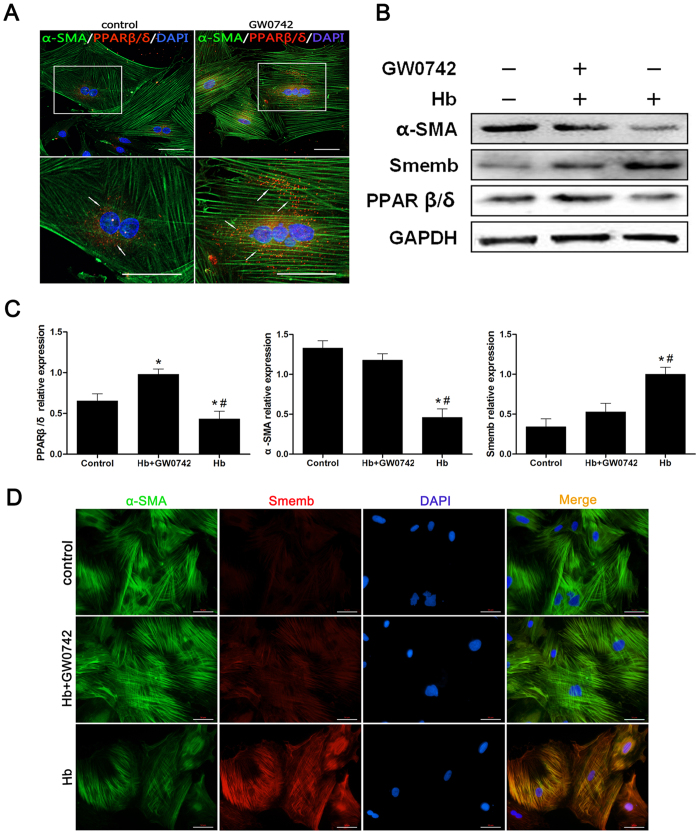
PPARβ/δ agonist GW0742 up-regulated PPARβ/δ expression and suppressed cerebral VSMC switch to synthetic phenotype. (**A**) Immunohistochemistry for α-SMA (green), PPARβ/δ (red) and DAPI (blue). (**B**) Representative image of PPARβ/δ, α-SMA and Smemb expression in cerebral VSMC at 24 hours after 10 μM Hb incubation with or without GW0742 pretreated respectively (n = 3 per group). (**C**) Representative quantitative analysis of PPARβ/δ, α-SMA and Smemb expression in cerebral VSMC at 24 hours after 10 μM Hb incubation with or without GW0742 pretreated respectively (n = 3 per group). (**D**) Immunohistochemistry for α-SMA (green), Smemb (red), and DAPI (blue) in cerebral VSMC at 24 hours after Hb incubation. All quantitative data were presented as mean ± standard deviation. Scale Bar = 50 μm. *P < 0.05 vs. Control; ^#^*P* < 0.05 vs. Hb + GW0742.

To ascertain whether the PPARβ/δ has any effects on phenotypic switch of VSMC, we attempted to promote the PPARβ/δ expression by agonist GW0742, and increased immunoreactivity of PPARβ/δ within VSMC was validated by immunocytochemistry (Fig. 2A). As shown in Fig. 2B,C there was a significant decrease in PPARβ/δ expression after hemoglobin incubation, which was reversed by GW0742 treatment. Moreover, GW0742 significantly enhanced the expression of α-SMA, while suppressed the Smemb expression even at the presence of hemoglobin (Fig. 2B,C). Consistently, immunocytochemical results showed increased intensity of α-SMA and decreased intensity of Smemb induced by GW0742 (Fig. 2D).

### Small Interfering-PPARβ/δ Inhibited the Expression of PPAR β/δ but Failed to Modulate Cerebral VSMC Phenotypic Switch

**Figure 3:**
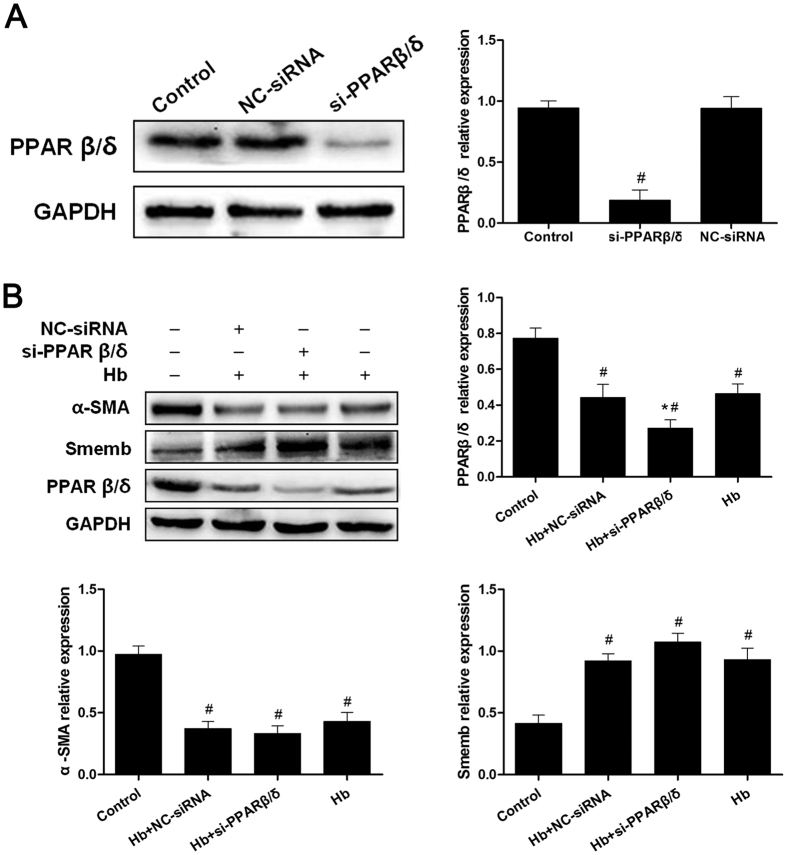
Small interfering-PPARβ/δ inhibited the PPAR β/δ expression but failed to modulate VSMC phenotypic switch. (**A**) Representative image and quantitative analysis of PPARβ/δ at 48 hours after transfection with siRNA in cerebral VSMC (n = 3 per group). (**B**) Representative image and quantitative analysis of PPARβ/δ, α-SMA and Smemb expression in cerebral VSMC at 24 hours after Hb incubation with or without si-PPARβ/δ treatment respectively (n = 3 per group). All data were presented as mean ± standard deviation. ^#^*P* < 0.05 vs. Control. *P < 0.05 vs. Hb + NC-siRNA.

To further validate the role of PPARβ/δ in VSMC phenotype switch following SAH, we negatively regulated PPARβ/δ expression with siRNA. As shown in Fig. 3A, the level of PPARβ/δ in cerebral VSMC was strongly suppressed by transfection of si-PPARβ/δ, whereas negative control siRNA (NC-siRNA) showed no significant effects (Fig. 3A,B). Those data suggested that si-PPARβ/δ effectively suppressed PPARβ/δ expression. After exposure to hemoglobin, however, no significant difference on α-SMA and Smemb expression was induced by PPARβ/δ decline (Fig. 3B).

### Overexpression of PPARβ/δ Induced by Ad-PPARβ/δ Prevented Cerebral VSMC Switch to Synthetic Phenotype

**Figure 4:**
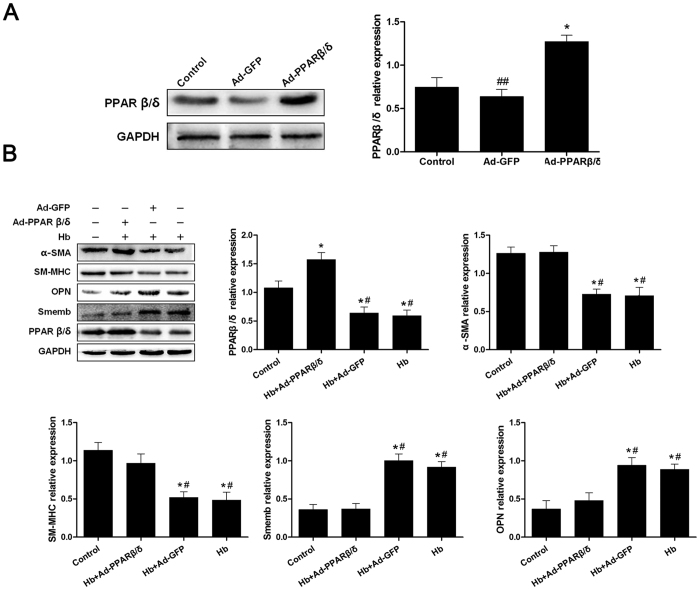
Overexpression of PPARβ/δ by Ad-PPARβ/δ Prevented Cerebral VSMC Switch to Synthetic Phenotype. (**A**) Representative image and quantitative analysis of PPARβ/δ at 48 hours after transfection with Ad-PPARβ/δ or Ad-GFP in cerebral VSMC (n = 3 per group). (**B**) Representative image and quantitative analysis of PPARβ/δ, α-SMA, SM-MHC, Smemb and OPN expression in cerebral VSMC at 24 hours after Hb incubation with or without Ad-PPARβ/δ pretreated respectively (n = 3 per group). All data were presented as mean ± standard deviation. *P < 0.05 vs. Control, ^##^*P* < 0.05 vs. Ad-PPARβ/δ; ^#^*P* < 0.05 vs. Hb + Ad-PPARβ/δ.

After transfection of adenovirus PPARβ/δ (Ad-PPARβ/δ) or adenovirus GFP (Ad-GFP) into VSMC respectively, immunoblot results showed that the Ad-PPARβ/δ markedly increased the PPARβ/δ expression with or without hemoglobin incubation (Fig. 4A,B). Moreover, Ad-PPARβ/δ administration significantly enhanced the expression of contractive proteins α-SMA and SM-MHC, while suppressing the expression of synthetic proteins Smemb and OPN as shown in Fig. 4B, attenuating the phenotypic switch by hemoglobin stimulation.

### PPARβ/δ Suppressed Cerebral VSMC Phenotypic Switch Partially through Activating PI3K/AKT Pathway Required Myocardin and SRF Nucleus Translocation

**Figure 5:**
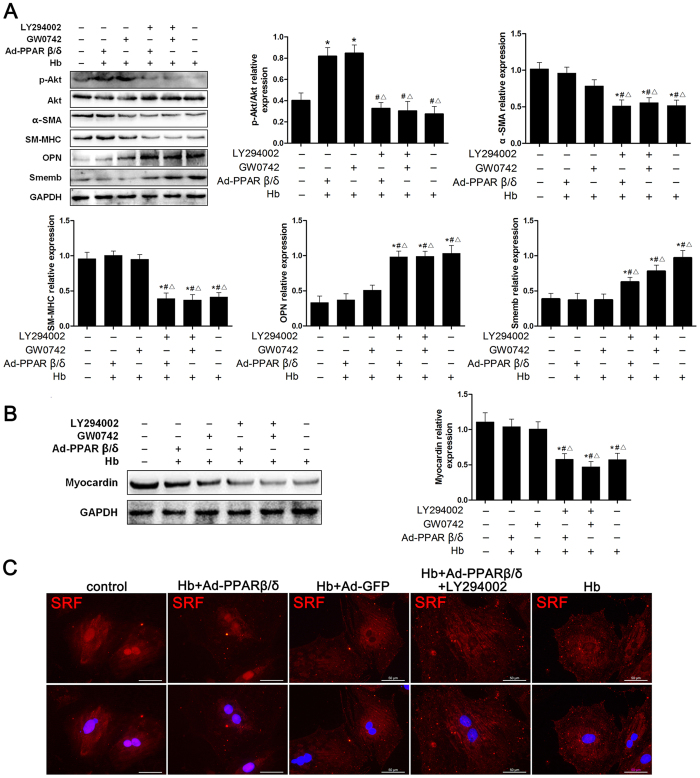
PPARβ/δ mediated cerebral VSMC phenotypic switch partially through PI3K/AKT pathway and promoted Myocardin expression and SRF nucleus translocation. (**A**) Representative image and quantitative analysis of p-Akt/Akt ratio, α-SMA, SM-MHC, OPN and Smemb expression in cerebral VSMC at 24 hours after Hb incubation with GW0742, Ad-PPARβ/δ or LY294002 treated respectively (n = 3 per group). (**B**) Representative image and quantitative analysis of Myocardin expression at 24 hours after Hb incubation with GW0742, Ad-PPARβ/δ or LY294002 treated (n = 3 per group). (**C**) Immunohistochemistry for SRF (red) and DAPI (blue) to determine SRF nucleus translocation. All quantitative data were presented as mean ± standard deviation. Scale Bar = 50 μm. *P < 0.05 vs. Control; ^#^*P* < 0.05 vs. Hb + Ad-PPARβ/δ; ^Δ^*P* < 0.05 vs. Hb + GW0742.

To evaluate the potential role of PI3K/AKT pathway in PPARβ/δ-induced phenotypic regulation of cerebral VSMC, we measured the Akt phosphorylation (p-Akt/Akt ratio) under different conditions. Immunoblotting results indicated no significant difference of Akt activation before and after hemoglobin treatment. However, both GW0742 and Ad-PPARβ/δ treatment induced significant increase in p-Akt level (Fig. 5A). Then LY294002 was used to inhibit the PI3K/AKT activity, and the effects of Ad-PPARβ/δ and GW0742 on VSMC phenotypic switch were abolished, as indicated by inhibited α-SMA and SM-MHC expression, as well as elevated Smemb and OPN level (Fig. 5A).

To seek the mechanism underlying PPARβ/δ function in VSMC phenotypic modulation, we determined the effects of PPARβ/δ on Myocardin expression and SRF nucleus translocation in VSMC. After hemoglobin incubation, the Myocardin expression within VSMC was dramatically suppressed. Ad-PPARβ/δ and GW0742 promoted Myocardin expression in the presence of hemoglobin, which was blocked by LY294002 (Fig. 5B). On the other hand, SRF nucleus translocation was impeded by hemoglobin treatment, and overexpression of PPARβ/δ by Ad-PPARβ/δ attenuated the effects of hemoglobin on SRF nuclear translocation. However, LY294002 prevented SRF nuclear translocation in spite of Ad-PPARβ/δ treatment (Fig. 5C).

### PPARβ/δ Regulated VSMC Phenotypic Switch and Vascular Remodeling after SAH, and Ameliorated Neurological Deficits

**Figure 6:**
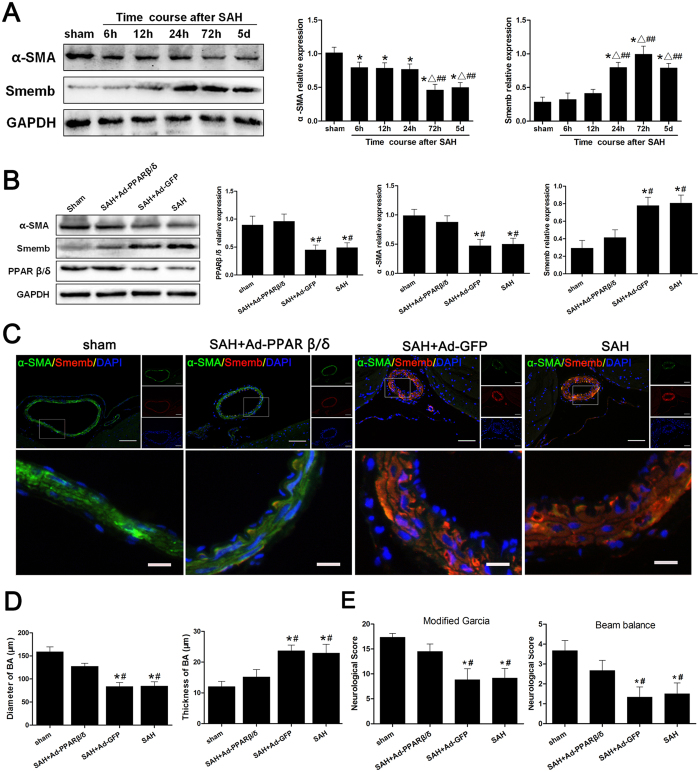
PPARβ/δ mediated cerebral VSMC phenotypic switch and vascular remodeling in SAH rats, and ameliorated neurological deficits. (**A**) Temporal profile and quantitative analysis of α-SMA and Smemb expression in the cerebral vessels within 5 days after SAH respectively (n = 6 rats per group). (**B**) Representative image and quantitative analysis of α-SMA, Smemb and PPARβ/δ expression in the cerebral vessels at 3 days after SAH after Ad-PPARβ/δ administration (n = 6 rats per group). (**C**) Immunohistochemistry of basilar artery with α-SMA (green), Smemb (red) and DAPI (blue) at 3 days after SAH after Ad-PPARβ/δ administration. (**D**) Quantitative analysis of the diameter and the wall thickness of basilar artery at 3 days after SAH after Ad-PPARβ/δ administration (n = 6 rats per group). (**E**) Modified Garcia test and beam balance in indicated groups at 3 days after SAH (n = 6 rats per group). All quantitative data were presented as mean ± standard deviation. Scale Bar = 50 μm (top) and 10 μm (bottom). *P < 0.05 vs. sham; ^Δ^*P* < 0.05 vs. 6 hours after SAH; ^##^*P* < 0.05 vs. 12 hours after SAH. ^#^*P* < 0.05 vs. SAH + Ad-PPARβ/δ.

To clarify the effects of PPARβ/δ on vascular remodeling after SAH, we established the endovascular perforation model to induce SAH in adult rats. The Smemb expression increased as early as 6 hours after SAH then peaked after 72 hours (Fig. 6A). Therefore, subsequent studies were performed at 72 hours post-SAH. In addition, the adenovirus Ad-PPARβ/δ was injected into the right lateral ventricle at 6 days before SAH. Immunoblot analysis revealed that PPARβ/δ expression increased with Ad-PPARβ/δ administration, however, α-SMA expression decreased within isolated basilar artery and circle of Willis arteries at 72 hours after SAH, accompanied by Smemb overexpression (Fig. 6B). Afterwards, the expression of α-SMA was significantly enhanced by Ad-PPARβ/δ treatment, while Smemb expression was inhibited (Fig. 6B). Consistently, the decreased intensity of α-SMA and increased intensity of Smemb was observed after SAH respectively, and Ad-PPARβ/δ reversed such histological changes (Fig. 6C). Furthermore, the thickness of vessel wall and lumen stenosis in basilar artery following SAH was significantly attenuated by Ad-PPARβ/δ treatment (Fig. 6D).

The mortality was not significantly different among the groups respectively (data not shown). As shown in Fig. 6E, SAH animals exhibited severe behavior deficits compared with sham animals. However, the Ad-PPARβ/δ + SAH group had a significant improvement in neurological function compared with the SAH group both in modified Garcia scale and beam balance test.

## Discussion

In the current study, we investigated the potential effects of PPARβ/δ on VSMC phenotypic switch and vascular remodeling following SAH both *in vivo* and *in vitro*. The main findings from our work includes: 1). Regulation of PPARβ/δ expression in cultured cerebral VSMC effectively alters its phenotypic switch after hemoglobin incubation; 2). PPARβ/δ suppresses cerebral VSMC phenotypic switch partially through activating PI3K/AKT pathway; 3). PPARβ/δ-induced PI3K/AKT activation contributes to Myocardin expression and SRF nuclear translocation; 4). PPARβ/δ regulates VSMC phenotypic switch and vascular remodeling in SAH rats, and alleviated neurological impairment.

Aneurysmal subarachnoid hemorrhage (aSAH) is a critical neurological disease with high morbidity and mortality^15^, and cerebral vasospasm was considered to mainly account for poor outcome associated with SAH^16^,^17^. Hemoglobin, as the hemolytic products of blood after SAH, has been reported to be responsible for delayed cerebral vasospasm. For example, Timothy E, *et al*. demonstrated that the hemoglobin contributes to excessive cerebral artery constriction following SAH^18^. After the incubation of hemoglobin with VSMC to mimic neuropathology of SAH, our results firstly revealed that hemoglobin pretreatment could also lead to cerebral VSMC phenotypic switch, causing dramatic downregulation of contractile proteins and overexpression of synthetic proteins in VSMC, consistent with the animal observation following SAH. Moreover, VSMC phenotypic switch peaked as early as 3 days post-SAH and lasted 2 days or even longer, which is highly similar to the temporal profile of cerebral vasospasm in patients^19^.

Recent studies indicated that OPN is reliable marker for synthetic VSMC^20^,^21^,^22^,^23^. In the present study, we found that the synthetic phenotype protein maker OPN increased after hemoglobin treatment in the VSMC. However, other researcher proved the recombinant exogenous OPN plays a role in stabilizing VSMC phenotype^24^. However, in that study no evidence showed whether recombinant exogenous OPN enters VSMC then serves to regulate vascular remodeling. It’s also possible that recombinant exogenous OPN interacts with other cell types (macrophage, endothelial cell also secrete OPN), and exerts indirect effects on VSMC. Thus the function of intracellular (VSMC) OPN remains unclear. In the current study we focused on OPN as a marker of synthetic phenotype rather than its function in vascular remodeling. Further work is needed to investigate its exact bioactivity.

As a major member of Peroxisome proliferator-activated receptors (PPARs) family^11^, PPARβ/δ was characterized to regulate the transcription of many target genes associated with vascular bioactivities and neuroinflammation^25^,^26^,^27^. For example, our previously work showed that PPARβ/δ alleviates early inflammatory response after SAH through blocking inflammation signaling^28^. Rikuta Hamaya, *et al*. reported the anti-inflammatory property of PPARβ/δ via attenuation of neointimal hyperplasia through suppressing VSMC proliferation^29^. In the current study, VSMC contractile-to-synthetic switch could be alleviated by application of Ad-PPARβ/δ or PPARβ/δ agonist GW0742, suggesting PPARβ/δ is necessary for maintaining the contractile state for VSMC homeostasis. The PPARβ/δ expression could be suppressed by hemoglobin treatment. Above conclusion was further supported by histological results in SAH animals. More intriguingly, the stenosis of basilar artery and thickening of the vessel wall was ameliorated by means of intracerebroventricular Ad-PPARβ/δ administration, meanwhile, there was a significant improvement in the neurological score at 3 days after SAH in the Ad-PPARβ/δ treatment group. Combining data showed that PPARβ/δ impedes VSMC phenotypic switch, and plays a protective role in vascular remodeling and neurological function after SAH.

However, further inhibition from siRNA failed to alter phenotypic switch in our research, implicating other potential mechanism to compensate the loss of PPARβ/δ function. Boerth *et al*. have shown that constitutively active catalytic domain of PKG within VSMCs results in increased expression of the contractile protein marker SM-MHC^30^. On the contrary, Brophy *et al*. reported that inhibition of PKG expression leads to a synthetic VSMC phenotype^31^. In addition, TGF-β has a well described ability to both inhibit proliferation and induce expression of contractile SMC marker genes^32^. Classically, TGF-β/Smad-3 has been associated with increased contractile marker gene expression via interaction with δEF-1^33^. Such results implicate the PKG or TGF-β/Smad maybe involved in this process to compensate the loss of PPARβ/δ function.

With regard to the mechanism underlying PPARβ/δ-induced VSMC phenotypic switch, PI3K/AKT signaling pathway was considered due to its association with VSMC function. Activated AKT was previously detected in vascular remodeling associated with VSMC anti-proliferation^34^. Then insulin-like growth factor could help VSMC maintain the differentiated phenotype via activation of the PI3K/AKT pathway^35^. These findings suggest that PI3K/AKT signaling may play an essential role in VSMC phenotypic regulation. In addition, Rosario Jime, *et al*. reported that the PPARβ/δ agonist GW0742 produces fast, dose-dependent relaxant effects in rat vascular tissue through PI3K/AKT pathway^36^. PPARβ/δ agonists can also activate PI3K/AKT signaling pathway in cardiac muscle cells for cardioprotection^37^. In the present study, the p-Akt level was elevated by Ad-PPARβ/δ and GW0742 treatment. However, LY294002, a potent inhibitor of PI3K/AKT activity, attenuated the effects of Ad-PPARβ/δ and GW0742 on hemoglobin-induced VSMC phenotypic switch, indicating that PI3K/AKT pathway participates in the PPARβ/δ regulation of VSMC phenotypic switch following SAH.

It has been well documented that Serum Response Factor (SRF) transferred to the nucleus to regulate VSMC differentiation and maintain its contractile phenotype binding with cis-acting elements CArG^38^,^39^. As a coactivator of the ubiquitous SRF transcription factor^38^, Myocardin is exclusively expressed in the smooth muscle (SMC) and cardiac muscle cell line^40^,^41^, activating CArG by forming a ternary complex with SRF, ultimately promoting the expression of specific contractile genes. On the contrary, when Dedicator of Cytokinesis 2 (DOCK2) inhibited Myocardin expression, blockage of SRF nucleus translocation led to the attenuation of contractile genes promoter activity^42^. Likewise, here the expression of Myocardin decreased in VSMC at 24 hours after hemoglobin incubation, accompanied by impeded SRF nuclear translocation. Xuehui Yang, *et al*. reported that Spry1 and Spry4 modulate SMC phenotype via regulation of PI3K/AKT activity and Myocardin expression^43^. In addition, interaction between Myocardin and PI3K/AKT can exert influence on phenotypic modulation of SMC^44^. The synthetic-to-contractile phenotypic transition in SMC was achieved by PPARβ/δ activation via endothelial release of Prostacyclin^45^. Here Ad-PPARβ/δ and/or GW0742 dramatically enhanced Myocardin expression and SRF nuclear localization, whereas LY294002 attenuated those positive effects from PPARβ/δ in VSMC, suggesting that PI3K/AKT activation by PPARβ/δ is responsible for Myocardin expression and SRF nuclear localization, contributing to the regulation of VSMC phenotypic switch.

In recent years, both basic and clinical evidence reported that changes in the microcirculation may be more important in relation to changes in perfusion and injury after SAH, whereas other studies advocated that unbalanced contractile/synthetic VSMC phenotype affected the size of the cerebral arteries and altered brain swelling or cerebral edema^4^,^24^,^46^. In the present study, we focus on the large artery rather than microvessel due to the enrichment of VSMC in large-middle artery. The results from our study validated the protective role of PPARβ/δ in VSMC phenotypic modulation, partially through the PI3K/AKT activation and downstream SRF nucleus localization, which benefits to the vascular remodeling following SAH. Further work should be done to evaluate whether PPARβ/δ would increase cerebral blood flow and prevent angiographic cerebral vasospasm. As a novel regulator of VSMC phenotype and vascular remodeling, PPARβ/δ may make more encouraging contribution to the therapeutic strategies of delayed cerebral ischemia following SAH.

## Materials and Methods

### Animal preparation

All experimental procedures were approved by the Animal Experiments Ethic Committee at the First Affiliated Hospital of Chongqing Medical University (Chongqing, China), and were performed in accordance with the Laboratory Animals in China and Guide for the Care. All rats were housed in specific pathogen-free conditions at the animal facilities at Animal Experimental Research Center of Chongqing Medical University. All surgeries were performed under anesthesia. Reporting of this study complies with the ARRIVE (Animal Research: Reporting *in vivo* Experiments) guidelines.

### Cell culture

The male Sprague-Dawley rat primary cerebral VSMCs of basilar arteries and circle of Willis arteries (ICell Bioscience, China) were cultured in dulbecco modified eagle medium and ham’s F-12 medium (DMEM/DF12, Gibco, USA, 1:1) supplemented with 10% fetal bovine serum (FBS, Gibco, USA) in 5% CO_2_ at 37 °C, and medium was replaced every 48 hours. Cells were confirmed by α-SMA (Abcam, United Kingdom, 1:200) and SM-MHC (Abcam, United Kingdom, 1:200) immunostaining. The cells with 85% confluence were subcultured. VSMCs less than 6 passages with 65% of confluence were used in the following experiments. To mimic SAH pathophysiology procedure and optimal stimulating concentration, cells were exposed to hemoglobin (Sigma, USA) at different concentrations ranged from 1 μM to 20 μM for 24 hours before following assays. After hemoglobin administration, cellular morphology was observed by inverted phase contrast microscope (Olympus, Japan) regularly, and western blots were used to detect the expression of α-SMA and Smemb in different concentrations groups.

### Cell Treatment

VSMCs were seeded in T-25 flasks or 6-well plates. First, Ad-PPARβ/δ and Ad-GFP were used to infect the cells for 48 hours when the cells were 65% confluence. The infection efficiency was monitor via fluorescence microscopy (Leica, Germany) by the means of expressed green fluorescent protein (GFP). Cells were then incubated with 10 μM hemoglobin for another 24 hours followed by other assays.

VSMCs were seeded into 6-well plates and were grown until 65% confluent, and were then transfected with 50 nM of si-PPARβ/δ (Ribobio, China) or negative control siRNA (NC-siRNA) using riboFECT^TM^ CP transfection reagents according to the manufacturer’s instructions (Ribobio, China). After 48 hours, the cells were incubated with 10 μM hemoglobin for another 24 hours. The protein expression and mRNA levels of PPARβ/δ were detected by western blot, quantitative real time-PCR and RT-PCR. More than 60% of the silence efficiency of the cells was accepted for all the experiments.

VSMCs were pre-incubated with 1 μM GW0742 (Selleck, USA) for 24 hours and then stimulated with 10 μM hemoglobin for additional 24 hours. To evaluate the involvement of PI3K/AKT in the effects of the PPARβ/δ on VSMC phenotypic switch, cells were pre-incubated with 1 μM LY294002 (Selleck, USA) 30 minutes before incubation with Ad-PPARβ/δ or GW0742, and all groups were incubated with 10 μM hemoglobin for another 24 hours followed by immunofluorescence and western blot.

### Immunocytochemistry

VSMCs were cultured on glass coverslips placed in 6-well plates at 65% confluence and pre-incubated Ad-PPARβ/δ or GW0742. For immunostaining, cells were rinsed with PBS thrice gently and fixed with 4% paraformaldehyde, and permeabilized with 0.01% Triton X-100 in PBS, then cells were blocked with 10% goat serum for 30 minutes at 37 °C. Subsequently, cells were incubated at 4 °C overnight with primary antibodies: mouse anti–α-SMA (Abcam, United Kingdom, 1:200), rabbit anti-PPARβ/δ (Abcam, United Kingdom, 1:200), goat anti–Smemb (Abcam, United Kingdom, 1:200) and rabbit anti–SRF (Abcam, United Kingdom, 1:200) followed by appropriate fluorescein isothiocyanate-conjugated and tetramethyl rhodamine isothiocyanate-conjugated secondary antibodies (Abbkine, USA, 1:200) for 1 hours at room temperature. Then, all cells were incubated with 4,6-diamidino-2-phenylindole (DAPI) for 15 minutes. Coverslips were mounted in antifade regent (Beyotime, China) and visualized by a fluorescence microscopy timely and effectively (Leica, Germany).

### Experimental Animals and SAH Model Induction

Male Sprague-Dawley rats (SD rats) weighing 300 to 350 g (8–10 weeks old) were used in this study, and the endovascular perforation model of SAH was implemented in rats as described previously^47^. Briefly, SD rats were anesthetized with 5% isoflurane, and then were positioned in a supine position. Expose the neck triangle formed by the sternohyoid/omohyoid, sternocleidomastoid and posterior belly of the digastric muscle. Discreetly blunt dissection the internal carotid artery (ICA), external carotid artery (ECA) and common carotid artery (CCA). Following, the ECA was coagulated and cut, a sharpened 4–0 monofilament nylon suture was inserted into the ICA from the ECA stump, and perforated the bifurcation of the ICA into middle cerebral arteries. The monofilament nylon suture was gently pushed forward until felt some resistance (8–20 mm), and then pushed further 1–2 mm to perforate the vessel. The suture was then withdrawn and the ICA was reperfused to produce SAH. Sham operated rats underwent the same procedures without perforation. As previous study^48^,^49^, neurological impairments were blindly evaluated using an 18-point score system known as the modified Garcia scale and another 4-point score system known as the beam balance test.

### Adenoviruses Administration

The total mortality of SAH in this study was 15.15% (15 of 99 rats). To verify the time course of the VSMC phenotypic switch after SAH, we randomly divided 36 SD rats into six groups with six rats in each group: sham, 6 hours after SAH, 12 hours after SAH, 24 hours after SAH, 72 hours after SAH, and 5 days after SAH. Western blots were used to detect the expression of α-SMA and Smemb in the cerebral vessels of each group.

Adenoviruses PPARβ/δ (Ad-PPARβ/δ), which was used to over-express PPARβ/δ, and adenoviruses GFP (Ad-GFP) as a control to Ad-PPARβ/δ (Genechem, China). Both of them were diluted to 1.3 × 10^10^ pfu/mL in enhanced transfection solution (Genechem, China) before were used. Intracerebroventricular injection procedure was performed as reported previously^50^. Briefly, rat’s head was fixed on the stereotactic frame, and then a small burr hole was drilled on the skull according to the coordinates of 1.0 mm posterior of bregma and 2.0 mm lateral of sagittal suture. The 25-guage needle was inserted into the left lateral ventricle through the burr hole, and 4.0 mm below the horizontal plane of bregma. Ten microliters diluted Ad-PPARβ/δ or Ad-GFP was then slowly injected into the lateral ventricle on the 6 days before SAH, so as to obtain the max amplification^28^. Thus, 24 rats were randomly divided into 4 groups for immunoblots of cerebral arteries: sham, SAH + Ad-GFP, SAH + Ad-PPARβ/δ and SAH group (n = 6/group). Another 24 rats were used for immunohistochemistry and neurological scores with similar distribution.

### Immunohistochemistry

Immunofluorescence staining for brainstem was performed on fixed frozen section to analysis the morphometric of basilar artery. Three days after SAH, SD rats were deeply anesthetized with 5% isoflurane and transcardially perfused with phosphate buffer saline (PBS) and 4% paraformaldehyde. After perfusion-fixation, the whole brain with the basilar artery was rapidly isolated from cranial cavity and immersed in the same fixative solution containing 20% sucrose at 4 °C for 24 hours, and then in 30% sucrose for another 48 hours. Coronal fixed frozen sections (10 μm) through the basilar artery were obtained by cryostat (Leica, Germany). Sections were fixed in the acetone 30 minutes and were then blocked with 10% goat serum for 2 hours at 37 °C. Subsequently, all fixed frozen sections were incubated at 4 °C overnight with primary antibodies: mouse anti–α-SMA (Abcam, United Kingdom, 1:200) and goat anti–Smemb (Abcam, United Kingdom, 1:200) followed by fluorescein isothiocyanate-conjugated and tetramethyl rhodamine isothiocyanate-conjugated secondary antibodies (Abbkine, USA, 1:200) for 2 hours at room temperature respectively. Cells nuclei were stained with 4,6-diamidino-2-phenylindole (DAPI) for 15 minutes. Fluorescence images were timely and effectively captured with the fluorescence microscope (Leica, Germany). The mean diameter and the thickness of basilar artery were calculated as described previously with slight modifications^51^,^52^. Briefly, for each vessel, three sequential sections (midpoint of the proximal, the middle and the distal) were taken, measured and averaged. The numerical value of micrometer per pixel was carried out by ascertainment of the scale, and the diameter and the thickness of basilar artery were measured using Image Pro-Plus software according to instructions. The inner perimeter of the vessels was measured by tracing the entire luminal surface of the intima, and the diameter (d) of the vessels was calculated according to the equivalent perimeter circle (d = measured inner perimeter/π). The thickness of the vessel wall was measured as the distance from the luminal surface of the intima to the outer border of the media at four different points of each artery. Those four measurements were averaged for one score.

### Western blot Analysis

The rats for protein determination were anesthetized and then killed by transcardially perfused. Afterward, with the aid of a microscope, the basilar arteries and circle of Willis arteries were carefully dissected free from each brain, cleared of connective tissue, and snap frozen in liquid nitrogen until were homogenized in RIPA buffer (Beyotime, China) containing protease inhibitors. Cultured VSMC in T-25 flasks following different treatments described above were washed thrice with PBS followed by protein extraction using RIPA buffer (Beyotime, China) containing protease inhibitors. The total protein concentration was measured using BCA Protein Assay Reagent (Beyotime, China).

The protein samples were denatured by boiling and were resolved (20 μg) on 6% or 10% sodium dodecyl sulfate polyacrylamide gels electrophoresis (SDS-PAGE) and then transferred onto polyvinylidene difluoridemembrane (PVDF, Bio-Rad, USA). The membranes were then blocked with 5% nonfat dry milk in Tris-buffered saline containing 0.1% Tween 20 (TBST) for 1 hour, and incubated overnight at 4 °C with specific antibodies: α-SMA (Abcam, United Kingdom, 1:200), SM-MHC (Abcam, United Kingdom, 1:500), Smemb (Abcam, United Kingdom, 1:200), OPN (Abcam, United Kingdom, 1:400), PPARβ/δ (Abcam, United Kingdom, 1:500), Myocardin (Abcam, United Kingdom, 1:400), p-Akt (Cell signal, USA, 1:1000), Akt (Cell signal, USA, 1:1000) and GAPDH (ProteinTech Group, China, 1:1000). Blots were subsequently incubated with relative horseradish peroxidase-conjugated IgG for 1 hour at 37 °C and visualized by using Chemiluminescence Kit (Beyotime, China) through X-ray film. Optical densities of these bands were quantified with Quantity One software 4.6.2. In all experiments, GAPDH was used as an internal reference. Three independent experiments were carried out to verify proteins expressions.

### Statistical Analysis

All *in vitro* experiments were performed with triplicate independent samples. All data were expressed as mean ± standard deviation (SD) and analyzed by SPSS 19.0. Chi-square test was used for the statistical analysis of neurological deficits between different groups. Mean group values were assessed using one-way ANOVA. A value of *p* < 0.05 was considered statistically significant.

## Additional Information

**How to cite this article:** Zhang, H. *et al*. PPARβ/δ, a Novel Regulator for Vascular Smooth Muscle Cells Phenotypic Modulation and Vascular Remodeling after Subarachnoid Hemorrhage in Rats. *Sci. Rep.* **7**, 45234; doi: 10.1038/srep45234 (2017).

**Publisher's note:** Springer Nature remains neutral with regard to jurisdictional claims in published maps and institutional affiliations.
